# Fractional transit compartment model for describing drug delayed response to tumors using Mittag-Leffler distribution on age-structured PKPD model

**DOI:** 10.1371/journal.pone.0276654

**Published:** 2022-11-04

**Authors:** Jong Hyuk Byun, Yunil Roh, In-Soo Yoon, Kwang Su Kim, Il Hyo Jung

**Affiliations:** 1 Department of Mathematics, College of Natural Sciences, Pusan National University, Busan, South Korea; 2 Department of Manufacturing Pharmacy, College of Pharmacy and Research Institute of Drug Development, Pusan National University, Busan, South Korea; 3 Department of Science System Simulation, Pukyong National University, Busan, South Korea; McGill University, CANADA

## Abstract

The response of a cell population is often delayed relative to drug injection, and individual cells in a population of cells have a specific age distribution. The application of transit compartment models (TCMs) is a common approach for describing this delay. In this paper, we propose a TCM in which damaged cells caused by a drug are given by a single fractional derivative equation. This model describes the delay as a single equation composed of fractional and ordinary derivatives, instead of a system of ODEs expressed in multiple compartments, applicable to the use of the PK concentration in the model. This model tunes the number of compartments in the existing model and expresses the delay in detail by estimating an appropriate fractional order. We perform model robustness, sensitivity analysis, and change of parameters based on the amount of data. Additionally, we resolve the difficulty in parameter estimation and model simulation using a semigroup property, consisting of a system with a mixture of fractional and ordinary derivatives. This model provides an alternative way to express the delays by estimating an appropriate fractional order without determining the pre-specified number of compartments.

## Introduction

Transit compartment models (TCMs) of perturbed tumor growth describe the delay process by which tumors are inhibited by drug administration [1–3]. The model generally consists of proliferating and damaged cell compartments. Apoptosis of the damaged cells by drugs does not occur immediately but with a delay. TCMs have been applied to pharmacokinetics and pharmacodynamics (PKPD) to explain the changes in tumors caused by drugs [4–6].

Logistic, exponential, and Gompertz models have been widely used to describe cell proliferation. Simeoni et al. propose a new growth model that increases exponentially at the beginning and linearly increases after the threshold [2]. A mortality process was commonly provided with the multiple of first-order degradation and drug concentration given by a pharmacokinetic model, or indirect response [7]. Subsequently, some proliferating cells enter the damaged phases given by the transit processes, and the number of the transit compartments determines the magnitude of delays. Existing TCMs considering transit processes explain cell-phase changes with the same mean residence time (MRT) [8, 9]. When the number of the transit compartments is *n*, the MRT of each phase is considered as 1/*k*_1_, indicating that *k*_1_ is a degradation rate from one compartment to another (total MRT *n*/*k*_1_). Such common TCMs can be modeled using Erlang distribution, called Erlang TCMs [10].

There are two considerations related to delays in the system of ODEs; the number of compartments (*n*) of the damaged cells and their degradation rate. Commonly, *n* is considered as hyperparameter [2, 4, 11]. This indicates that *n* is pre-specified for the simulation and then other parameters are estimated using simulation for fitting the experimental data. Our study aims at an alternative way to capture the tumor delay after drug administration. This approach resolves the hyperparameter problem and enables simultaneously estimating parameters to fit the data. For this purpose, we use a system based on a fractional derivative for describing the delay instead of the system of ODEs.

A study for the physical and geometric interpretation of fractional derivatives is shown in [12]. The fractional derivative is used to model biological phenomena and obtain applications in this field. The applications to biological models constructed by fractional-order differential equations produce more realistic results compared to their integer-order counterparts [13, 14]. These studies describe that fractional derivatives involve memory and are considerably advantageous for working with biological processes. However, they do not compare integer-order models against experimental data. Therefore, our study constructs a fractional-based TCM model and compared changes in drug delay with ODE-based TCM.

In this paper, we present a fractional TCM based on the Caputo fractional derivative. An age-structured model is first formulated and a Mittag-Leffler (ML) distribution, a non-Markovian distribution, is used for describing the delay [15]. Age is regarded as the time when the cells enter the damaged cells after the drug injection. Consequently, the damaged cells are modeled using an age-structured model and the mortality rate of the damaged cells depends on both age and drug concentration. Mortality rate of damaged cells is formulated using the convolution of the mortality function *k*_*out*_(*C*, *u*) of proliferating cells *u* and a density function *f* based on age *a*.

Among density functions, we apply one of the non-Markovian distributions to ML distribution, unlike phase-type distributions including Erlang distribution. The ML distribution is of the form 1 − *E*_*α*_(−*t*^*α*^), where $E_{\alpha }\left(t\right)=\sum_{n=0}^{\infty }$ *t*^*n*^/Γ(1 + *α* ⋅ *n*) for *α* ∈ (0, 1] [16]. The ML distribution is an exponential distribution for *α* = 1 and a heavy-tailed distribution for 0 < *α* < 1. This distribution is related to a fractional derivative that generalizes integer-order derivatives to allow for arbitrary-order derivates [17, 18].

We utilize Laplace transform to obtain the simple form because the density function of ML distribution is given by a series. The resulting system is consisting of ordinary and fractional derivative equations that are equations with fractional-order derivatives. In contrast to common TCMs (or Erlang TCMs) given by a system of ODEs to describe the transition of the damaged cells, the fractional transit compartment model (fractional TCM) has a form of a single equation and its mortality is given by a fractional-order derivative. Unlike the pre-specified number of compartments in Erlang TCMs, fractional TCM may resolve the problem of determining the number of compartments and flexibly expresses the delay by estimating an appropriate fractional order. This model is separated into two parts: a delay expressed by the fractional derivative and an ODE equation describing the dynamics of the delayed damaged cells.

The fractional TCM is compared to the Erlang TCM in which the growth rate of proliferating cells is given by Simeoni et al. [2]. To compare them, the simulation of the fractional TCM is carefully considered because fractional TCM has a system of equations with a mixture of ordinary and fractional derivatives in an equation. The property of semigroup of a fractional derivative is applied to the model under some conditions to resolve this challenge [19, 20]. Subsequently, model robustness and sensitivity analysis were investigated for model validation. We explored the change of parameter values based on the amount of data, showing that data fitting using fractional TCM is applicable.

The remainder of this paper is organized as follows. In the next section, fractional TCM is derived from the two-compartment model using ML distribution. The PK model, considering dosing regimens, is applied to the model. Model robustness and sensitivity analysis are investigated. This model is compared with Erlang TCM after parameter estimation and particularly, delays are compared with the impacts of the parameter change. In the Discussion section, we summarize the findings and compare the results with previous studies. In the Methods section, we formulate a compartment model of proliferating and damaged cells for describing delayed tumor dynamics.

## Results

### Derivation of Erlang TCM

The system induced from the method section is summarized as follows:

$$\begin{array}{cc} \frac{du}{dt}= & k_{in}\left(u,w\right)-k_{out}\left(C,u\right)\\ \frac{dy}{dt}= & k_{out}\left(C,u\right)-\left(k_{out}*f\right)\left(t\right), \end{array}$$ (1)

associated with *u*(0) = *u*_0_, $y\left(t\right)=\int_{0}^{\infty }\phi \left(a,t\right)da$, and *w*(*t*) = *u*(*t*) + *y*(*t*). Herein, *f* is a density function depending on age *a*, and *u* and *y* represent proliferating and damaged cells, respectively. *k*_*in*_ and *k*_*out*_ are the growth and mortality functions of the proliferating cells, respectively. The operator * represents convolution. We assumed that no drug administration indicated that all cells are proliferating, so *y*(0) = 0. That is, cells begins to be damaged after a drug is administrated. This assumption follows from the Magni et al. [4].

For example, if *f* is from the point distribution (Dirac-delta function) in Eq (1), that is, *f*(*a*) = *δ*(*a* − *T*), then

$$\begin{array}{c} \frac{dy}{dt}=k_{out}\left(C\left(t\right),u\left(t\right)\right)-k_{out}\left(C\left(t-T\right),u\left(t-T\right)\right), \end{array}$$

which represents a delay differential equation (DDE) [21–23]. This equation indicates that all individuals had the same residence time *T*. As the other example, we present an explicit system of ODEs on density function *f* using Erlang distribution. This system of ODEs is called Erlang TCM that describes the transition process from one compartment to another. Some of tumor cells enter the damaged phases once a drug is administered. If they cascade a multiple-step process with a chain of compartments, it demonstrates the delays motivated by the pathway of signal transduction [8]. We considered an age density of *f*_*n*_ in place of *f* to formulate Erlang TCM from Eq (1), and *f*_*n*_ was given by

$$\begin{array}{c} f_{n}\left(a\right)=k_{1}\cdot \frac{{\left(k_{1}a\right)}^{n-1}}{(n-1)!}e^{-k_{1}a}, \end{array}$$

which is a density of Erlang distribution. We have the following relations using the linear chain trick because *f*_*n*_ is differentiable [24, 25].

$$\begin{array}{cc} \frac{df_{1}\left(t\right)}{dt} & =-k_{1}f_{1}\left(t\right)\\ \frac{df_{n}\left(t\right)}{dt} & =k_{1}\left(f_{n-1}-f_{n}\left(t\right)\right),\;n\geq 2. \end{array}$$

If we define *E*_*n*_ as *E*_*n*_(*t*) = (*k*_*in*_ * *f*_*n*_)(*t*)/*k*_1_, *n* ≥ 2, then by differentiating *E*_*n*_, we have the following system of ODEs as follows:

$$\begin{array}{cc} \frac{du}{dt} & =k_{in}\left(u,w\right)-k_{out}\left(C,u\right)\\ \frac{dy}{dt} & =k_{out}\left(C,u\right)-k_{1}E_{n}\left(t\right)\\ \frac{dE_{n}}{dt} & =k_{1}\left(E_{n-1}\left(t\right)-E_{n}\left(t\right)\right), \end{array}$$

provided with *E*_*i*_(0) = 0, *i* = 1, 2, ⋯, *n*. If *y*_*i*_ is the damaged tumor cells with age *i*, then the total damaged cells *y* can be considered as *y* = *y*_1_ + *y*_2_ + ⋯ + *y*_*n*_. Let *y*_*i*_ = *E*_*i*_. Then, Erlang TCM can be derived as

$$\begin{array}{cc} \frac{du}{dt} & =k_{in}\left(u,w\right)-k_{out}\left(C,u\right)\\ \frac{dy_{1}}{dt} & =k_{out}\left(C,u\right)-k_{1}y_{1}\left(t\right)\\ \frac{dy_{2}}{dt} & =k_{1}\left(y_{1}\left(t\right)-y_{2}\left(t\right)\right)\\ \vdots \\ \frac{dy_{n}}{dt} & =k_{1}\left(y_{n-1}\left(t\right)-y_{n}\left(t\right)\right), \end{array}$$ (2)

satisfying

$$\begin{array}{c} dy/dt=d\left(y_{1}+y_{2}+\cdots +y_{n}\right)/dt=k_{out}\left(C,u\right)-k_{1}E_{n}. \end{array}$$ (3)

**Fig 1:**
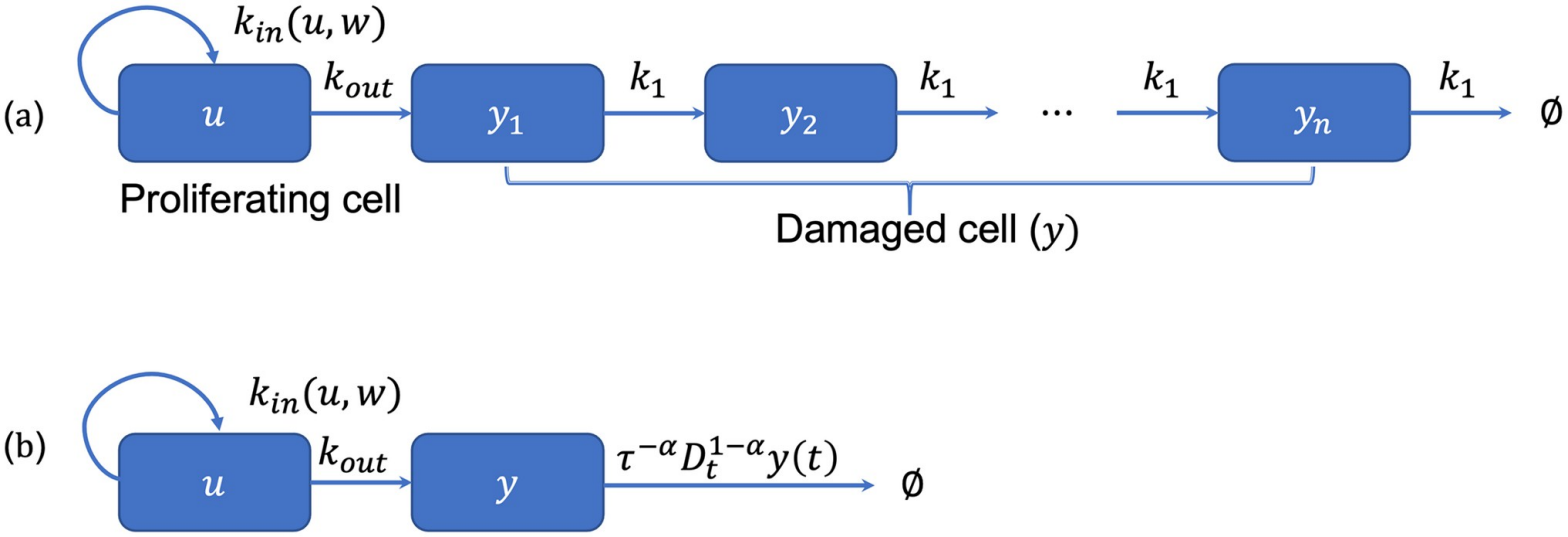
Schematic diagram of TCMs modeled by distributions. (a) Erlang TCM. Total tumor cells are given by *w* = *u*+ *y*_1_ + ⋯ + *y*_*n*_. (b) Fractional TCM. Total tumor cells are given by *w* = *u* + *y*.

Fig 1(a) shows the schematic diagram of Erlang TCM.

### Derivation of fractional TCM

A density function *f* of ML distribution is used for deriving fractional TCM. Since *f* has the form of series, it is difficult to find the closed form, resulting in analysis intractable and computationally expensive. To resolve these difficulties, Laplace transformation can be applied to Eq (1) to obtain a simple form that results in a form of fractional derivative equation. For this work, we consider a survival function *S*(*t*) given by

$$\begin{array}{c} S\left(t\right)=E_{\alpha }(-(t/\tau {)}^{\alpha }), \end{array}$$

*τ* > 0 and density function *f* is given by *f*(*t*) = −*dS*/*dt*. Taking the Laplace transform of *S* and *f* from *t* to *s* gives

$$\begin{array}{c} L\left(S\left(t\right)\right)=1/\left(s(1+{\left(\tau s\right)}^{-\alpha })\right) \end{array}$$

and

$$\begin{array}{c} L\left(f\left(t\right)\right)=1-sL\left(S\left(t\right)\right)={\left(\tau s\right)}^{-\alpha }/\left(1+{\left(\tau s\right)}^{-\alpha }\right), \end{array}$$

provided with (*τs*)^*α*^ < 1. We define a kernel *K*(*t*) by

$$\begin{array}{c} L\left(K\left(t\right)\right)=L(f(t))/L(S(t))=\tau^{-\alpha }s^{1-\alpha }. \end{array}$$

To connect *K*(*t*) and Laplace transform of (*k*_*in*_ * *f*)(*t*), we utilize a fractional derivative. The process is summarized as:

(i) Define Riemman-Liouville (RL) fractional integral operator of *α* ∈ [0, 1] as for *α* > 0,

$$\begin{array}{c} J_{t}^{\alpha }y\left(t\right)=\frac{1}{\Gamma (\alpha )}\int_{0}^{t}{\left(t-s\right)}^{\alpha -1}y\left(s\right)ds \end{array}$$

and for *α* = 0, $J_{t}^{\alpha }=I$, identity operator.

(ii) The RL derivative is defined as for 0 < *α* ≤ 1,

$$\begin{array}{c} {{}^{RL}D_{t}}^{\alpha }y\left(t\right)=\frac{d}{dt}J_{t}^{1-\alpha }y\left(t\right)=\frac{d}{dt}\{\frac{1}{\Gamma (1-\alpha )}\int_{0}^{t}\frac{y(s)}{{\left(t-s\right)}^{\alpha }}ds\}, \end{array}$$

and for *α* = 0, set ${{}^{RL}D_{t}}^{\alpha }=I$, identity operator. Notably, by definition, RL derivative with order 1 satisfies

$$\begin{array}{c} {{}^{RL}D_{t}}^{1}y=\frac{d}{dt}J_{t}^{0}y\left(t\right)=\frac{d}{dt}y\left(t\right)=y^{\prime }\left(t\right). \end{array}$$

(iii) The Caputo fractional derivative is defined as for 0 ≤ *α* ≤ 1,

$$\begin{array}{c} D_{t}^{\alpha }y\left(t\right)=\frac{1}{\Gamma (1-\alpha )}\int_{0}^{t}\frac{y^{\prime }\left(s\right)}{{\left(t-s\right)}^{\alpha }}ds \end{array}$$

for 0 < *α* ≤ 1 or for *α* = 0, set $D_{t}^{\alpha }=I$, identity operator. By using Taylor expansion for fractional derivative [26], RL and Caputo fractional derivative have the following relationship:

$$\begin{array}{c} D_{t}^{\alpha }y\left(t\right)={{}^{RL}D_{t}}^{\alpha }\left(y\left(t\right)-y\left(0\right)\right), \end{array}$$

for 0 ≤ *α* ≤ 1. This yields $D_{t}^{1}y\left(t\right)=y^{\prime }\left(t\right)$ if *y*(0) = 0. Additionally, the Laplace transform of the Caputo derivative [18] has the form of

$$\begin{array}{c} L(D_{t}^{1-\alpha }y\left(t\right))=s^{1-\alpha }L\left(y\left(t\right)\right)-s^{-\alpha }y\left(t\right){|}_{t=0}=s^{1-\alpha }L(y\left(t\right)), \end{array}$$

provided by *y*(0) = 0. This equation reveals the relation between Laplace transform of Caputo derivative and ordinary derivative. Now let a function *G* given by *G*(*t*) = (*k*_*in*_ * *f*)(*t*). From Eq (1) associated with *S*(*t*), we have Laplace transform of

$$\begin{array}{c} L\left(y\left(t\right)\right)=L\left(k_{in}\right)\cdot L\left(S\left(t\right)\right), \end{array}$$

and

$$\begin{array}{c} L\left(G\left(t\right)\right)=L\left(k_{in}\right)\cdot L\left(f\left(t\right)\right). \end{array}$$

This enables the determination of the Laplace transform of *G*(*t*) by

$$\begin{array}{c} L\left(G\left(t\right)\right)=L\left(K\left(t\right)\right)\cdot L\left(y\left(t\right)\right)=\tau^{-\alpha }s^{1-\alpha }\cdot (\frac{L(D_{t}^{1-\alpha }y\left(t\right))}{s^{1-\alpha }})=\tau^{-\alpha }L(D_{t}^{1-\alpha }y\left(t\right)). \end{array}$$

Therefore, we have $G\left(t\right)=\tau^{-\alpha }\cdot D_{t}^{1-\alpha }y\left(t\right)$ as taking the inverse Laplace transform. Thus, fractional TCM is induced as follows.

$$\begin{array}{cc} \frac{du}{dt}= & \;k_{in}\left(u,w\right)-k_{out}\left(C,u\right)\\ \frac{dy}{dt}= & \;k_{out}\left(C,u\right)-\tau^{-\alpha }D_{t}^{1-\alpha }y\left(t\right), \end{array}$$ (4)

provided with *u*(0) = *w*_0_, *y*(0) = 0. We restrict the range of *α* by 0 < *α* < 1 for the computational purpose because the system returns to the system of ODEs if *α* = 0 or 1. The second equation in Eq (4) shows that delays of the damaged cells are described by a fractional order and the computation of a fractional-order derivative in time involves an integration over the entire time history of the function (Fig 1(b)). There are many numerical simulation methods for the fractional models, but there are no available methods to implement the system consisting of the mixture of ordinary and fractional derivatives to the best of our knowledge [27–29]. To perform the simulation, we need an additional semigroup property and then the Eq (4) is transformed into the simple form to conduct the simulation. We will discuss this property detail in the other subsection.

### Growth and mortality functions of the proliferating cells

Simeoni et al. develop the rate change of the proliferating cells *u* given by a perturbed growth (tumor) function *k*_*in*_(*u*, *w*) and mortality function *k*_*out*_(*C*, *u*) based on first-order, commonly used in Erlang TCMs [2]. *k*_*in*_ and *k*_*out*_ are given by

$$\begin{array}{c} k_{in}\left(u,w\right)=\frac{\lambda_{0}u}{{\left(1+{\left(\frac{\lambda_{0}}{\lambda_{1}}w\right)}^{\phi }\right)}^{\frac{1}{\phi }}}\;\text{and}\;k_{out}\left(C,u\right)=\eta \cdot C\cdot u. \end{array}$$

For a sufficiently large *ϕ* (more than 10), *k*_*in*_ has approximately exponential growth with a linear rate *λ*_0_*u* less than or equal to the threshold *w*_*th*_ = *λ*_1_/*λ*_0_ and nonlinear growth with a rate of *λ*_1_ ⋅ *u*/*w* otherwise. They provide two growth rates in a single form for computational reasons.

### Pharmacokinetic model

The PK model is from the Magni study [4], which also used the growth function given by Simeoni et al., and consists of two compartments, as follows:

$$\begin{array}{cc} \frac{dq_{1}}{dt} & =-k_{01}q_{1}\left(t\right)-k_{21}q_{1}\left(t\right)+k_{12}q_{2}\left(t\right)+v\left(t\right)\\ \frac{dq_{2}}{dt} & =k_{21}q_{1}\left(t\right)-k_{12}q_{2}\left(t\right)\\ C(t) & =\frac{q_{1}\left(t\right)}{V}, \end{array}$$ (5)

where *q*_1_ and *q*_2_ are the quantity of drug in the plasma and peripheral compartments, respectively, and *V* is the volume in plasma. *C*(*t*)(*ng* ⋅ *ml*^−1^) is the concentration, and *v*(*t*) is the bolus administration (*ng* ⋅ *kg*^−1^). The obtained drug concentration *C* is applied to Erlang and fractional TCMs shown in Eqs (2) and (4).

### Parameter values and estimations

**Fig 2:**
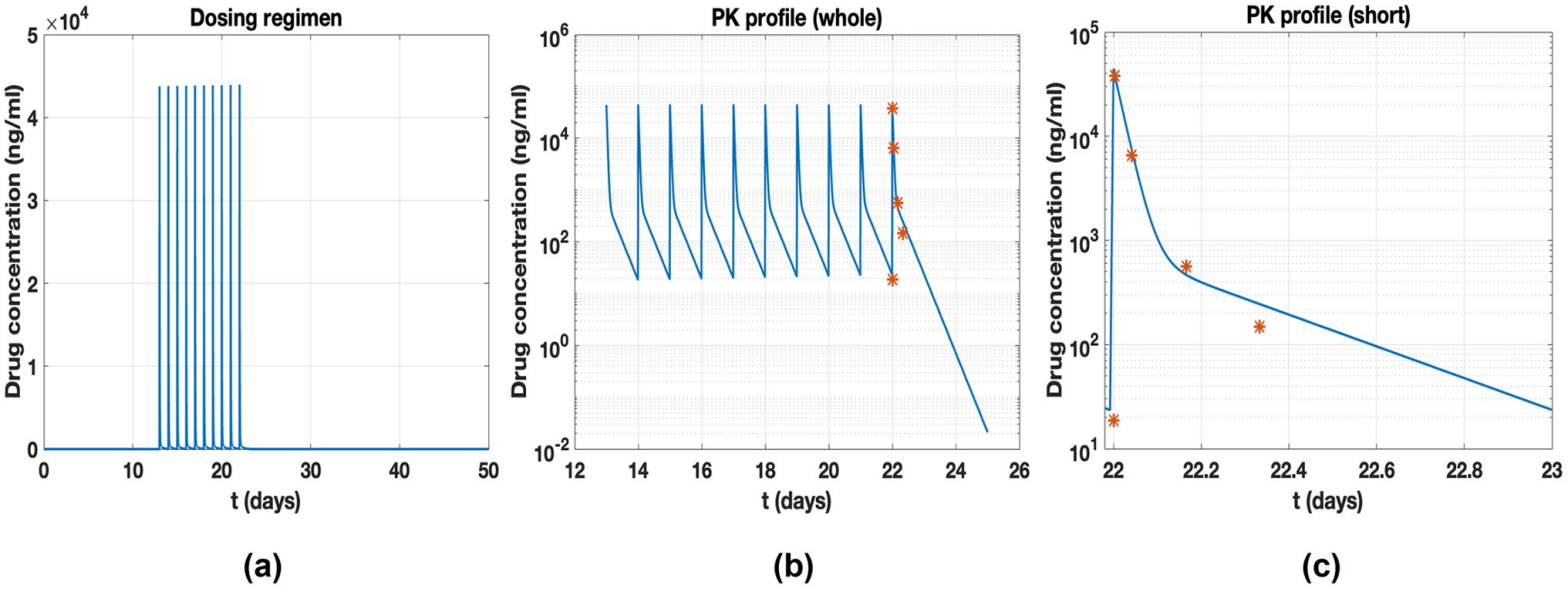
(a) Dose regimen per day from day 13–22. (b) Simulated PK profiles are plotted. The red symbols indicate observed PK data after the final injection. (c) Simulated PK profile shown in the study [4] is reproduced.

The tumor data consists of ten points of tumor size versus time profile, as shown in *mouse 150* [4]. The tumor is implanted on day 0 with an initial tumor size *w*_0_ given by 0.0121*g*, and the drug is administered on day 13 and injected every day for ten days. Their study and PK profile are reproduced in detail, as shown in Fig 2. Parameter values from the PK model are given by *k*_01_ = 1.6, *k*_21_ = 0.2353, *k*_12_ = 0.1699 and *V* = 1028 and for per injection time *t*_*in*_, *q*_1_(*t*_*in*_) = 4.5 ⋅ 10^7^, where *t*_*in*_ is dose timing for the ten injections. The parameter values related to the growth rate *k*_*in*_ are *λ*_0_ = 0.25, *λ*_1_ = 0.4603, and *ϕ* = 20.

The other parameter values are estimated. Data fitting of the tumor size using Erlang and fractional TCMs is performed using estimated parameters as follows. *η* = 0.37847 and *k*_1_ = 0.54379 for Erlang TCM, and *α* = 0.945, *η* = 0.24657 and *τ* = 3.6852 for fractional TCM. Objective functions for optimization of parameters are given by nonlinear least-squares. Model optimization and simulations are conducted using Matlab 2021a. Simulation for Erlang TCM is conducted using ODE45 based on the Runge-Kutta method and fde12 for simulating fractional TCM [27]. A fractional derivative equation solver can be used after a modification with the semigroup property.

### System modification for implementation: Semigroup property

Eq (4) is a fractional system consisting of two equations. One of the equations has ordinary and fractional derivatives. In this case, it is challenging to simulate the system. Particularly, fde12 cannot be directly applied because the system consists of a mixture of ordinary and fractional derivatives in an equation. To resolve this problem, we considered a latent variable *z* such that *z* is defined as $z=D_{t}^{1-\alpha }y$. Generally, the Caputo derivative dose not satisfy a semigroup property with respect to *α* of differentiation; however, it possesses a semigroup property under some additional assumptions [19, 20], which are $D_{t}^{1}y\left(t\right)$ and $D_{t}^{1-\alpha }\left(D_{t}^{\alpha }y\left(t\right)\right)$ are continuous on **R**. Then semigroup property

$$\begin{array}{c} y^{\prime }\left(t\right)=D_{t}^{1}y\left(t\right)=D_{t}^{\alpha }\left(D^{1-\alpha }y\right) \end{array}$$

is satisfied. The system given by Eq (4) is transformed into a multi-order system of fractional-order equations and computer simulation such as fde12 can be used. The resulting system is as follows.

$$\begin{array}{ll} \frac{du}{dt}= & \frac{\lambda_{0}u}{{\left(1+{\left(\frac{\lambda_{0}}{\lambda_{1}}w\right)}^{\phi }\right)}^{\frac{1}{\phi }}}-\eta \cdot C\cdot u\\ D_{t}^{1-\alpha }y= & z\\ \frac{dy}{dt}= & \eta \cdot C\cdot u-\tau^{-\alpha }z, \end{array}$$ (6)

where *C* is from the PK model (Eq (5)). Notably, the damaged cells (*y*) given by *z*, are delayed.

### Comparison between Erlang and fractional TCMs

**Fig 3:**
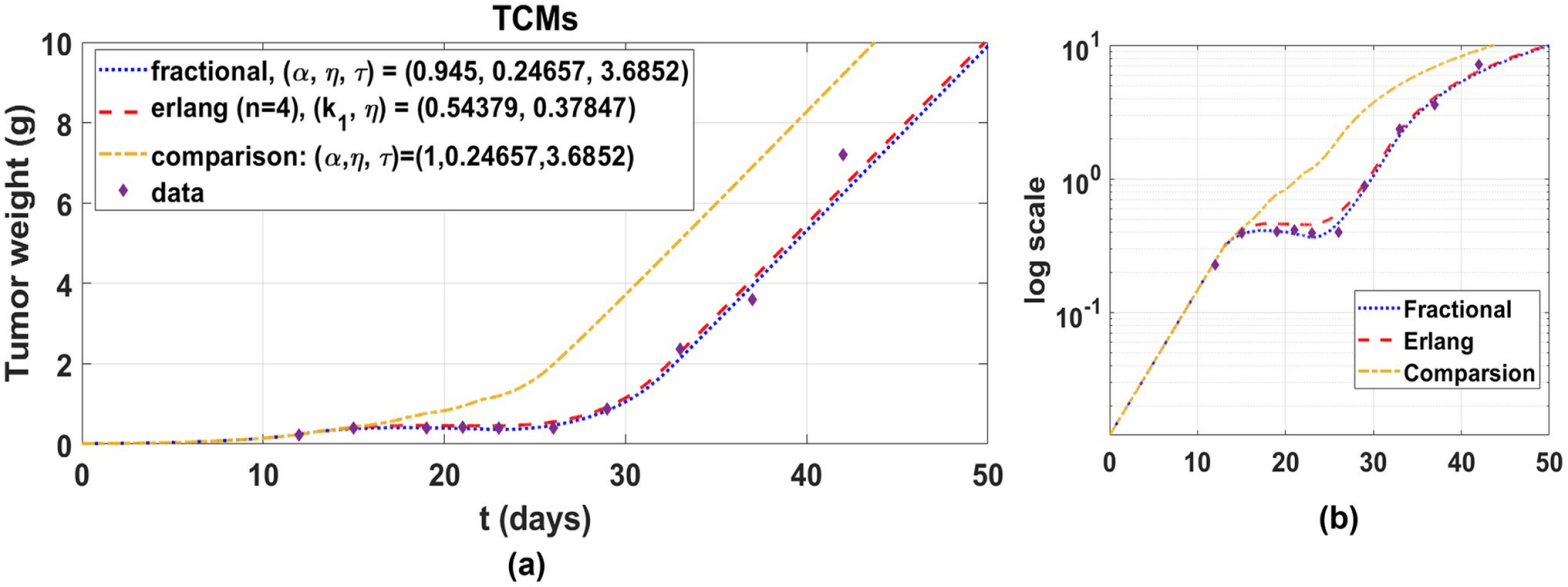
Comparison of Erlang and fractional TCMs. Scheduled drugs are administered ten times after day 13. The pre-specified number of damaged cells is *n* = 4. RMSE is calculated to find AIC. (a) We observe the delay effect of fractional order *α*, *α* = 1 (no delay). (b) Log-scale is used to compare data fit quality.

Erlang and fractional TCMs given by Eqs (2) and (6) are compared after parameter estimations, as shown in Fig 3. Erlang TCM consists of five compartments. One of the compartments represents the proliferating cells and the others indicate the damaged cells in transition processes. We plot the curves in log-scale to see delays during the drug effect phase over day 13. We investigate delays based on the number of compartments in Erlang TCM. Root mean square errors (RMSE) is calculated, given by

$$\begin{array}{c} (\sum_{n=1}^{N}{\left(D_{n}-\left(w_{n}\right)\right)}^{2}/N{)}^{1/2}, \end{array}$$

to determine the optimal number of compartments. Here, *N* is the amount of data, *D*_*n*_ is the *n*th data, and *w*_*n*_ = *u*^*n*^ + *y*^*n*^. Furthermore, *u*^*n*^ and *y*^*n*^ are model outputs of proliferating and total damaged cells at the *n*th data time. The number is four, which represents four transit compartments, except for the proliferating compartment. Fractional TCM has RMSE = 0.3392 which is comparable to Erlang TCM, which has RMSE = 0.3139 for full data *N* = 10. From the obtained RMSE, we measure Akaike information criterion (AIC) which is an estimator of prediction error and thereby relative quality of statistical models for data. AIC is given by

$$\begin{array}{c} N\cdot (ln(2\pi )+1)+2N\cdot ln(\text{RMSE})+2(k+1) \end{array}$$

and *k* as number of parameters. AIC of Erlang and fractional TCMs are 11.2023 and 14.7538 which are comparable. We note that although the AIC of Erlang TCM is smaller than fractional TCM, the number of compartments for describing delays of the damaged cells is pre-specified to simulate the model. This shows that Erlang TCM is necessary for two parameters *k*_1_ and *n* for capturing the delays, but *n* is pre-specified through several tests and then *k*_1_ can be estimated. In the case of fractional TCM, however, there are two estimated parameters *τ* and *α* to describe the delays, but the estimation simultaneously enables. Moreover, the number of equations is smaller in the fractional TCM. Our approach enables reduce the difficulty to specify *n* and provides a single equation for the delayed dynamics of the damaged cells.

### Model robustness

**Fig 4:**
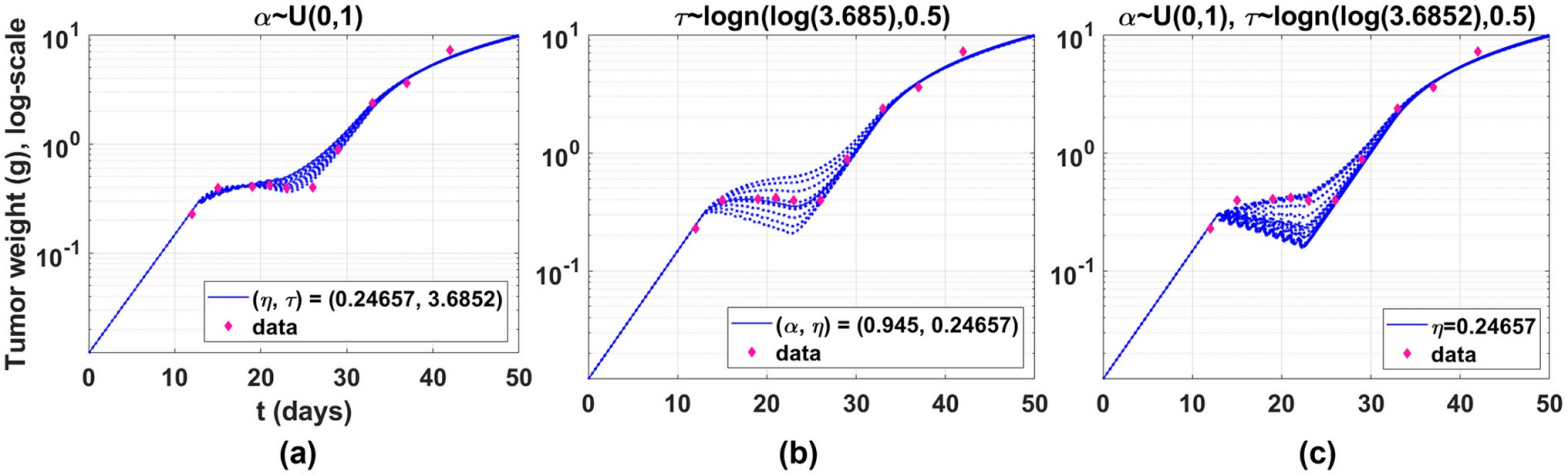
Delays of fractional TCM upon variations of *α* and *τ*. (a) Ten points of *α*’s are selected from the uniform distribution. Delay is larger when *α* is smaller. (b) Ten points of *τ*’s are selected from the lognormal distribution with mean log(3.685) and variance 0.5. Delay is larger when *τ* is larger. (c) Twenty points are randomly selected from the uniform and lognormal distributions. The model accurately captures various delays caused by drug injections. Additionally, the model shows that tumors begin to increase after stopping doses.

In the two TCMs, we perform data fitting from partial data for model robustness. Model robustness is that if the outputs or forecasts are consistently accurate even if one or more of the input variables or assumptions are drastically changed due to unforeseen circumstances. We conduct the model robustness test only for the parameters related to the delays. A change of delay is explored by varying two parameters *α* and *τ*. We do not investigate the perturbation of *η* because this is already investigated in Erlang TCM. *α* and *τ* are randomly selected from uniform and lognormal distributions. Ten (*α*, *τ*) or twenty (both) samples are plotted in Fig 4. The other parameters are the same as we mentioned before. In Fig 4(a), delays are smaller when *α* is larger. Conversely, delays are larger when *τ* is larger, as shown in Fig 4(b). In Fig 4(c), *α* and *τ* are simultaneously changed. The results demonstrate that fractional TCM can capture various delays for multiple dosing. Furthermore, the fractional model describes dynamics that tumors begin to increase when dosing is stopped on day 23.

### Change of parameter estimation based on the amount of data

**Fig 5:**
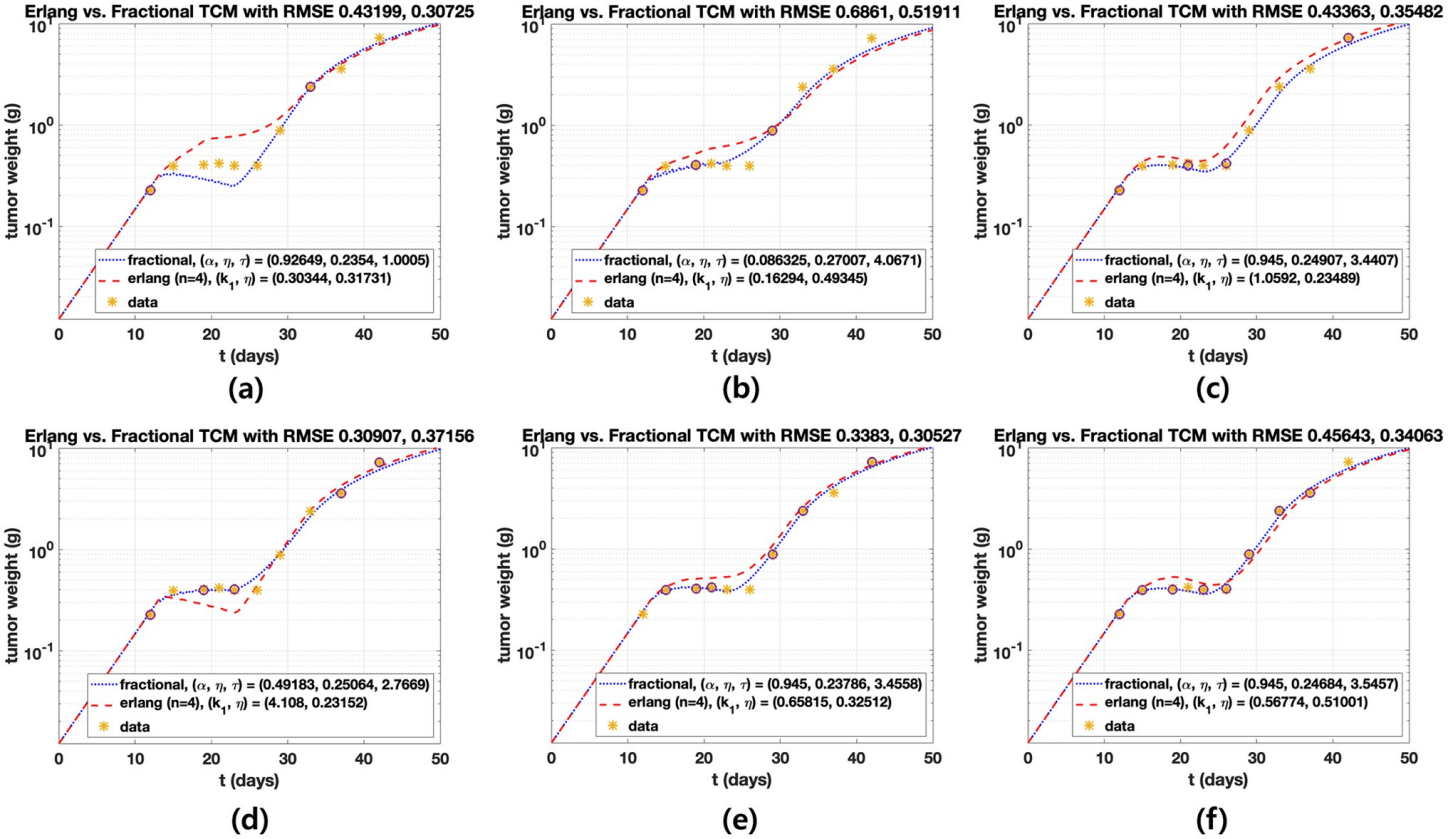
Parameter estimations are conducted according to the amount of data. log-scale about *y* axis is used. The circle represents test data (Points 2, 3, 4, 5, 6, 8) and the star shapes represent full data (ten points). The number of compartments is four in Erlang TCM, except for the compartment of proliferating cells. Samples are randomly selected among full data once the amount of data was selected. (a) Both models fail to predict full data. (b) Both TCMs accurately fit the data; however, Erlang TCM exhibits less suppression of tumor during treatment in this dataset. (c), (e), (f) Both models accurately fit the data. In (d), Erlang TCM exhibits stronger tumor suppression during treatment in this dataset but data fit quality is unreliable.

**Fig 6:**
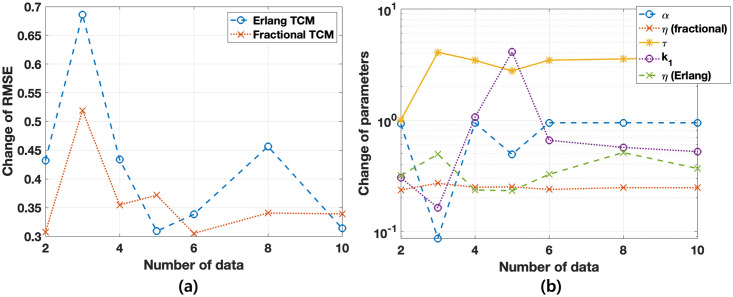
Changes in the parameter values and RMSE based on the amount of data. (a) RMSE is smaller as the amount of data increases. (b) Parameters converge as the amount of data increases.

We test to find minimal data points to capture the full data set using the model. This work shows how the estimated parameters with fewer data points are close to them obtained by the full data set and proposes minimum data points for the given phenomenon. To that end, we randomly selected two, three, four, five, six and eight points (circle) among ten data points (star), as shown in Fig 5. To compare predictions of the two models, *α*, *η*, and *τ* are estimated in fractional TCM, and *k*_1_ and *η* with *n* = 4 are estimated in Erlang TCM according to the number of data. Initial and parameter ranges are given by (*k*_1_, *η*) = (0.4, 0.1) from [0, 0] to [10, 1] for Erlang TCM and (*α*, *η*, *τ*) = (0.93437, 0.1, 1) from [0.01, 0, 1] to [0.95, 10, 10] for fractional TCM. Other parameters are the same as mentioned in previous sections. Model predictions for the two points of data are observed in Fig 5(a), and both models fail to predict full data. Other cases, except for two points, fairly predict full data with less data set. However, Erlang TCM is suppressed over data during the treatment, as shown in (d). We also calculate the change of RMSE and values of the parameters based on the amount of data, as shown in Fig 6. RMSE is smaller when the number of data increases. Moreover, the values of the parameters become stable and the values converged. Thus, parameters estimated by randomly selected six data points are similar to those composed of the full data set, meaning that the fractional TCM fairly captures the full data set.

### Sensitivity analysis

**Fig 7:**
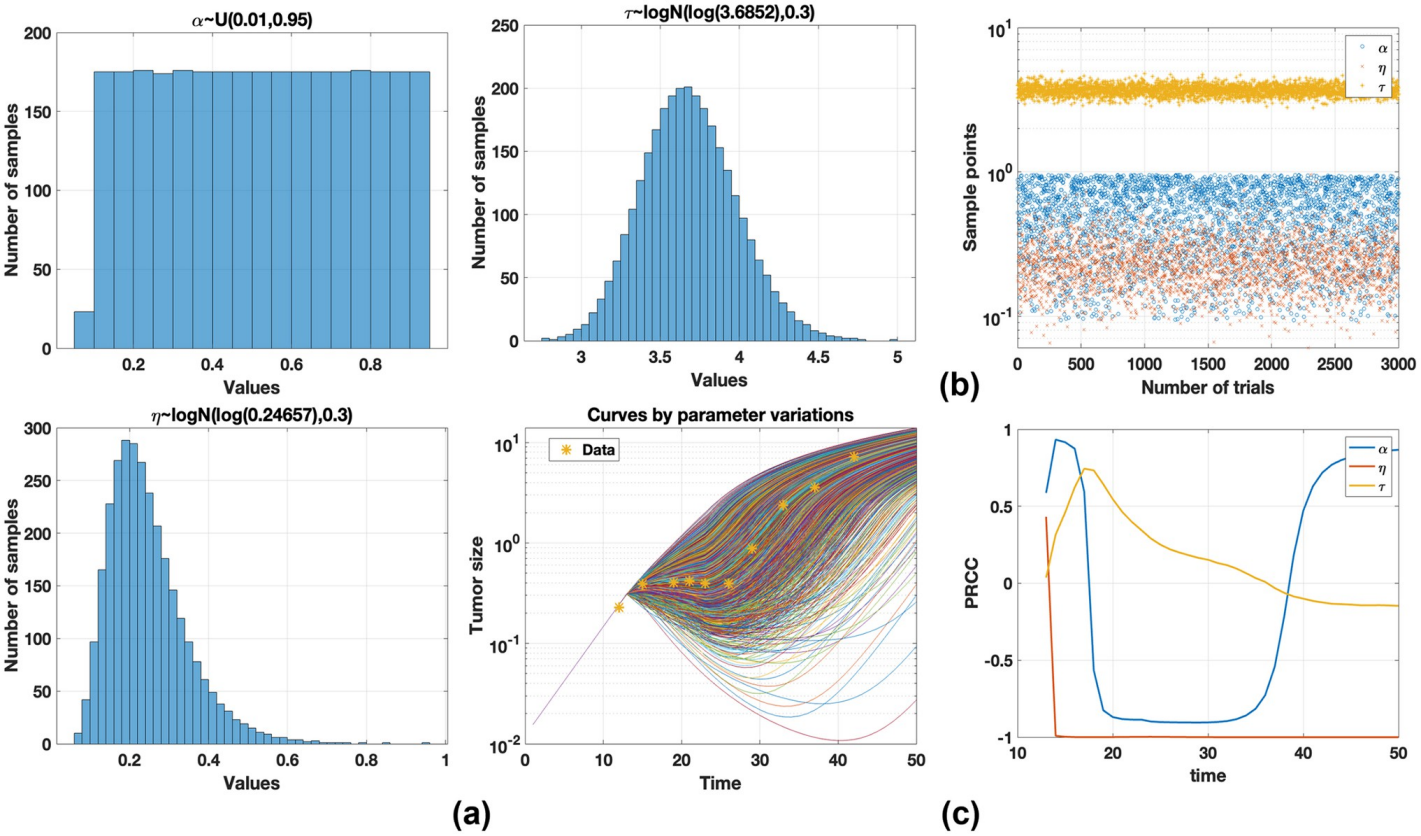
(a) *α*, *η*, and *τ* are selected from the uniform and lognormal distributions, respectively. Three thousand samples obtained using the LHS method are selected and some of the corresponding curves are plotted. (b) Sampled points are shown. (c) From sampled points, we perform PRCC. *η* is negative when tumor size increases. *τ* has a negative influence after day 37 but it has a small PRCC. *α* has positive and negative influences on tumor size over time. From this analysis, *α* and *τ* are important parameters with respect to the change in tumor size.

Sensitivity analysis is performed to understand parameter influences of the tumor size in fractional TCM. Partial rank correlation coefficient (PRCC) is investigated for global sensitivity. PRCC is the correlation between two variables after removing the effect of variables. Detailed explanation and formula are seen in [30]. PRCC in this study is measured for the relation among tumor sizes and parameters. We select 3000 samples of *α*, *τ*, and *η* using the Latin hypercube sampling (LHS) method to perform PRCC, as shown in Fig 7(a). The obtained sample points of parameters are plotted in Fig 7(b). The values of PRCCs are calculated and plotted over time in Fig 7(c). Before day 13 (no injection), the parameters do not influence tumor size. *η* has a fully negative influence on the increase of tumor size. *τ* has a strong positive influence during injections; however, the relation decreases and has negative influence as time elapses. *α* has a negative influence, but it changes positively. From this analysis, *α* and *τ* are important parameters with respect to the change of tumor sizes with drug administration, and we may control these parameters in this model.

## Discussion

Responses of cell populations have attracted attention for use in PKPD. TCMs describe delays or aging processes in cell populations after drug administration. TCMs explain delays in which damaged cells are eliminated as transit processes and are usually expressed as an ODE system. We present fractional TCM to describe the delay process. A stochastic process is applied to the transit process by a convolution of the density function and degradation rate. Using Mittag–Leffler distribution and Laplace transformation, we formulate the fractional TCM. This model consists of two equations: proliferating and damaged cells. The latter consists of both ordinary and fractional derivatives that are related to the delay of tumor size after drug administration.

Model simulation is performed and compared to Erlang TCM. We utilize the semigroup property for model implementation. Thus, the system consists of three compartments: proliferating cells, delay process given by fractional derivative, and damaged cells. Parameter estimation, robustness, parameter change by the amount of data, and sensitivity analysis are performed to verify the model’s quality and usefulness. Similar to Erlang TCM, fractional TCM can be used to describe tumor delay caused by drugs without the pre-specified number of damaged compartments with fewer compartments. This approach has a benefit that enables data estimation simultaneously and alleviates the difficulty of choosing the number of compartments *n* for the damaged cells.

Our simulation study has some limitations associated with the determination of the time step and fractional order. In simulation implementation, the time step should be sufficiently small. In this study, the time step is 0.0039 (that is, 2^−8^). Otherwise, model predictions hardly demonstrate data set greater than the given time step, even if parameters are correctly selected. The reason could be the numerical method or fractional order. The calibration of the time step for the simulation is not the main issue in the Erlang TCM but is necessary for the fractional TCM. If the time step is too small, then computational work is expensive, and in the opposite case, the model simulation could be unreliable. Another limitation is fractional order *α*. In contrast to Erlang TCM which has the parameter *k*_1_ related to mean duration and the number of compartments *n*, the physical meaning of *α* is still ambiguous. We utilize *α* for describing the delay in this study. This study does not determine which models are better; however, fractional TCM may be applicable when the number of compartments for the delay is unknown or data fitting seems unreliable. We present an alternative model for TCM and provide a simple model modification for the implementation of simulation using the semigroup property in the system.

Erlang TCMs are widely used in PKPD [2, 4, 8], epidemics [31–33], and other research areas [11, 34, 35]. However, the time delay is discretely described with regards to the number of compartments [36]. To describe delays, fractional models are substituted with a fractional derivative instead of the ordinary derivative on the left-hand side of the equation, which explains the abnormal kinetic (power-law kinetic) [37–39]. Additionally, the use of the fractional models on the left-hand side is already used in the PK study, demonstrating the fat-tail end behavior of the drugs [40–42]. In the SIR model, a model using a stochastic process is proposed to reflect the delay of infected individuals [17, 43]. Our model approach proposes a method similar to that of the SIR model to represent the delay of cell apoptosis due to drugs, considering data fitting. Some studies have a single equation with the mixture of ordinary and fractional derivatives, or a system of fractional derivatives only in the equations. However, our system is different because the model is a system consisting of a mixture of fractional and ordinary derivatives in an equation.

## Materials and methods

### Mathematical model formulation: Age-structured-based perturbed tumor model using survival function

We consider a cohort of cells: a group of cells of age in an interval of length Δ*a*. We obtain the Mckendrick-von Foerster equation as follows.

$$\begin{array}{cc} \frac{du}{dt} & =k_{in}\left(u,w\right)-k_{out}\left(C,u\right)\\ \frac{\partial \phi }{\partial t}+\frac{\partial \phi }{\partial a}\cdot \frac{da}{dt} & =-\mu (a,C)\phi (a,t), \end{array}$$ (7)

where *u* = *u*(*t*) is the number of proliferating cells with initial *u*(0) = *u*_0_ and *C* = *C*(*t*) is the drug concentration. The total number of damaged cells at time *t* is given by $y\left(t\right)=\int_{0}^{\infty }\phi \left(a,t\right)da$. *w* = *w*(*t*) is the total number of cells given by *w*(*t*) = *u*(*t*)+ *y*(*t*). We expected that the unit of age is the same as that of time and so, we assumed *da*/*dt* = 1. Additionally, if the mortality rate *μ*(*a*, *C*) depends only on age *a*, then *μ*(*a*, *C*) = *μ*(*a*) [44]. This assumption is reliable because the age depends on the duration after the drug *C* is injected. At *a* = 0, the boundary condition is *ϕ*(0, *t*) = *k*_*out*_(*C*, *u*), indicating that damaged tumor cells of age zero are created by tumor size and drug concentration. The initial condition of *ϕ* is given by *ϕ*(*a*, 0) = 0, indicating that all tumors without drug administration are proliferating cells. Under these conditions, time *t* is the time since administration of the drug.

The existence and uniqueness of the solution for Eq (7) are proven using the method of characteristics, which can be solved as follows [45].

$$\begin{array}{c} \phi \left(a,t\right)=\{\begin{array}{c} k_{out}\left(C\left(t-a\right),u\left(t-a\right)\right)e^{\int_{0}^{\infty }\mu \left(\alpha \right)d\alpha },\;\;t\geq a\\ 0,\;\;t<a, \end{array} \end{array}$$ (8)

which holds that

$$\begin{array}{c} \phi \left(a,t\right)=k_{out}\left(C\left(t-a\right),u\left(t-a\right)\right)e^{\int_{0}^{\infty }\mu \left(\alpha \right)d\alpha } \end{array}$$

for all *t* ≥ 0. Additionally, we integrated the second equation in Eq (7) over the age *a*. Therefore,

$$\begin{array}{c} \frac{dy}{dt}=k_{out}\left(C,u\right)-\int_{0}^{\infty }\mu \left(a\right)\phi \left(a,t\right)da. \end{array}$$ (9)

The mortality rate *μ*(*a*) is related to the probability of survival. We consider a stochastic process to verify it. Let *S*(*a*) = *Pr*{*T* ≥ *a*} be the probability of survival from zero to age *a*. *S*(*a*)*y* denotes the cells remaining in the cohort until age *a*;

$$\begin{array}{c} S(a+\Delta a)y-S(a)y=-\mu (a)S(a)y\Delta a \end{array}$$

denotes the number of cells that die in Δ*a*. Subsequently, both sides are divided by *y*Δ*a* and Δ*a* → 0,

$$\begin{array}{c} \frac{dS}{da}=-\mu \left(a\right)S\left(a\right). \end{array}$$

Because the initial value of *S* is the probability of survival until age 0, we assume *S*(0) = 1. By solving this equation, we obtain $S\left(a\right)=exp\left(-\int_{0}^{a}\mu \left(\alpha \right)d\alpha \right)$, whose derivative defines the probability density function *f* of *Pr*{*T* < *a*}, such that *f*(*a*) = −*dS*/*da* = *μ*(*a*)*S*(*a*). This indicates the probability that a cohort dies within age *a*. Thus, *μ* can be interpreted as the death-hazard rate. By substituting Eq (8) into Eq (9) with *f*, we derive the following equation.

$$\begin{array}{ccc} \frac{dy}{dt} & = & k_{out}\left(C,u\right)-\int_{0}^{\infty }k_{out}\left(C\left(t-a\right),u\left(t-a\right)\right)f\left(a\right)da\\ & = & k_{out}\left(C,u\right)-\left(k_{out}*f\right)\left(t\right), \end{array}$$ (10)

where * denotes the convolution operator. Thus, we obtain the master equation for the perturbed tumor model as follows.

$$\begin{array}{ccc} \frac{du}{dt} & = & k_{in}\left(u,w\right)-k_{out}\left(C,u\right)\\ \frac{dy}{dt} & = & k_{out}\left(C,u\right)-\left(k_{out}*f\right)\left(t\right), \end{array}$$ (11)

associated with *u*(0) = *u*_0_, $y\left(t\right)=\int_{0}^{\infty }\phi \left(a,t\right)da$, and *w*(*t*) = *u*(*t*) + *y*(*t*). The mortality (elimination) rate of collection of damaged cells *y* is delayed and given in the form of convolution.

## Supporting information

S1 Data (ZIP)
